# Must Keep up with the Times

**Published:** 1885-09

**Authors:** 


### Must Keep up with the Times.

A doctor was lately brought before the German tribunals for having neglected to keep himself informed as to the modern methods of practice. A servant who received a wound in the chest in April last died from septicæmia under the care of this doctor, who, despising antiseptic dressings, treated his patient according to ancient usages. The court held that "every practitioner should keep himself informed in the accomplished progress of science, and have an exact knowledge of modern systems of treatment. If these had been employed the patient's life might have been saved, hence the liability for negligence."

